# Innate immunity in rickettsial infections

**DOI:** 10.3389/fcimb.2023.1187267

**Published:** 2023-05-09

**Authors:** Andrés F. Londoño, Diana G. Scorpio, J. Stephen Dumler

**Affiliations:** ^1^ The Henry M. Jackson Foundation for Advancement in Military Medicine, Bethesda, MD, United States; ^2^ Department of Pathology, School of Medicine, Uniformed Services University of the Health Sciences, Bethesda, MD, United States; ^3^ Host-Pathogen Interactions Program, Texas Biomedical Research Institute, San Antonio, TX, United States

**Keywords:** rickettsia, anaplasma, innate immunity, toll-like receptors, autophagy, inflammasome

## Abstract

Rickett**s**ial agents are a diverse group of alpha-proteobacteria within the order Rickettsiales, which possesses two families with human pathogens, Rickettsiaceae and Anaplasmataceae. These obligate intracellular bacteria are most frequently transmitted by arthropod vectors, a first step in the pathogens’ avoidance of host cell defenses. Considerable study of the immune responses to infection and those that result in protective immunity have been conducted. Less study has focused on the initial events and mechanism by which these bacteria avoid the innate immune responses of the hosts to survive within and propagate from host cells. By evaluating the major mechanisms of evading innate immunity, a range of similarities among these bacteria become apparent, including mechanisms to escape initial destruction in phagolysosomes of professional phagocytes, those that dampen the responses of innate immune cells or subvert signaling and recognition pathways related to apoptosis, autophagy, proinflammatory responses, and mechanisms by which these microbes attach to and enter cells or those molecules that trigger the host responses. To illustrate these principles, this review will focus on two common rickettsial agents that occur globally, *Rickettsia* species and *Anaplasma phagocytophilum*.

## Introduction

Rickettsial pathogens are among a broad range of obligate intracellular bacteria that have evolved through the combined colonization and expansion in arthropod vectors and vertebrate hosts. Although defined by genome similarities, the overall Rickettsiales class that is comprised of several families possesses only two families with established pathogens of humans, Rickettsiaceae and Anaplasmataceae. The nature of arthropod-borne transmission, whether by the bites of ticks or mites, *via* the inoculation of louse- or flea bite wounds with infected arthropod feces, or *via* ingestion of infected foods, provides an accelerated mechanism to bypass key host innate protections, in particular, the skin barrier. However, despite this, a range of pathogens with similar genomes have a variable capacity to infect and to initiate disease in humans and in animal models. These aspects of innate protection or evasion thereof, are further impacted by the effects of the arthropod vectors, such as with the saliva of ticks or mites. These various pathogens that require infection of living cells in their hosts adhere to, invade, alter, replicate in, evade immune recognition, and facilitate host cell injury and disease that varies by pathogen and host. Understanding these events is key to defining approaches toward disease control. This broad area of investigation requires a more comprehensive review than possible in this review, which will thus focus on salient issues relating to innate immune response either of hosts and the evasion mechanisms used by members of the *Rickettsia* genus as examples of cytosolic pathogens, and by *Anaplasma phagocytophilum* as an example of an intravacuolar pathogen, representing the two most domestically common human infections by rickettsiae (**Table 1**).

**Table 1:** Key innate immune events and mechanisms in diseases caused by *Rickettsia* spp. and *Anaplasma phagocytophilum* infection.

*Rickettsia* spp. Infection Event	Important features	References
Early events in *Rickettsia* transmission and dissemination	• Transmission by ticks, mites, fleas, or lice bypasses host mechanical barriers	
	• Infection of CD68+ cells in dermis	(Walker et al., 1999)
	• Initial spread *via* lymphatics to blood and tissues	(Fournier et al., 2005)
	• Suppressive response with a high frequency of Foxp3^+^ T regulatory cells	(Fang et al., 2007)
	• Alter macrophage transcriptional programs	(Curto et al., 2019a; Curto et al., 2019b)
Rickettsial adhesion and invasion events	• Cell receptors and proteins - Ku70, α2β1 integrin, FGFR1, and Epac1	(Martinez et al., 2005; Gong et al., 2013; Hillman et al., 2013; Sahni et al., 2017)
	• Rickettsial adhesins/invasins	(Chan et al., 2009)
	• Clathrin and caveolin-2 in rickettsial endocytosis and internalization	(Chan et al., 2009)
	• Rickettsial phospholipases and hemolysins in endosomal escape	(Sahni et al., 2019)
Innate immunity triggering events	• TLR4, TLR2, and NLRP3 signaling	(Jordan et al., 2008; Quevedo-Diaz et al., 2010; Smalley et al., 2016)
	• Suppression of CD14 by pathogenic species	(Curto et al., 2019b; Kristof et al., 2021)
	• Autophagy and its subversion	(Bechelli et al., 2019; Engström et al., 2019; Bechelli et al., 2021; Engström et al., 2021)
Animal models to study innate immunity	• C57BL/6 knockout mice such as *Mhc1^-/-^*, *Prf1^-/-^*, *Ifng^-/-^*, or *Nlrp3^-/-^*	(Osterloh, 2017)
	• C3H/HeJ mice	(Jordan et al., 2008)
	• AG129 mice	(Burke et al., 2021)
***Anaplasma phagocytophilum* Infection Event**	Important features	
*A. phagocytophilum* adhesion and invasion	• Bacterial AipA, Asp14, OmpA, AnkA	(Naimi et al., 2020)
	• Host PSGL-1/sLe^x^ tetrasaccharide, Abi-1, WAVE complex	(Goodman et al., 1999; Lin et al., 2007)
	• Autophagosomes and autophagy subversion	(Truchan et al., 2013; Truchan et al., 2016a; Truchan et al., 2016b)
*A. phagocytophilum* reprogramming of neutrophil functions	• Cytokine responses and proinflammatory response	
	• IFNγ, IL10 and IL8	(Dumler et al., 2000; Martin et al., 2000)
	• Protracted degranulation	(Choi et al., 2004a)
	• Regulation of microbial detection	(Garyu and Dumler, 2005; Garyu et al., 2005)
	• Suppression of antimicrobial responses	
	• Diminished emigration of infected neutrophils	(Choi et al., 2003; Park et al., 2003)
	• Inhibition of respiratory burst	(Banerjee et al., 2000; Choi and Dumler, 2003; Thomas et al., 2005)
	• Defective phagocytosis and microbial killing	(Whist et al., 2002; Garyu et al., 2005)
	• Delayed apoptosis	(Choi et al., 2005; Ge and Rikihisa, 2006)
	• Epigenetic reprogramming by AnkA	(Garcia-Garcia et al., 2007; Garcia-Garcia et al., 2009b)
Innate immune recognition	• TLR2 and bacterial lipoproteins	(Choi et al., 2004b)
	• NLRC4 inflammasome activation	(Chen et al., 2012; Wang et al., 2016a; Wang et al., 2016b)
	• NF-κB activation	(Choi et al., 2004b; Dumler et al., 2020a)
	• IFNγ and Stat1 signaling	(Akkoyunlu and Fikrig, 2000; Choi and Dumler, 2013; Choi et al., 2014)
	• NK and NKT lymphocyte cytotoxicity defects	(Choi et al., 2007; Scorpio et al., 2018)
Severe disease syndromes	• Mild vs. severe (cytokine hyperproduction and immune discoordination) disease outcomes Macrophage activation syndrome (MAS) and hemophagocytic lymphohistiocytosis (HLH), sepsis, acute respiratory distress syndrome	(Dumler et al., 2007; Camacci et al., 2018a; Rocco et al., 2020)
Animal models to study innate immunity	• *Ifng^-/-^, IL10^-/-^, Stat1^-/-^*, A129 (*Ifna/br^-/-^*), AG129 (*Ifna/b/gr^-/-^)*, NKT and NK cell deficient, TLR, inflammasome, inflammatory signaling (*Myd88^-/-^, Cybb^-/-^, Asc* ^-/-^ *, AnxA2^-/-^, Rip2^-/-^*).	(Martin et al., 2001; Walker et al., 2001; Von Loewenich et al., 2004; Wang et al., 2004; Birkner et al., 2008; Wang et al., 2016a; Burke et al., 2021; Müller et al., 2021)

## The genus *Rickettsia*

### Early events in *Rickettsia* transmission and dissemination

*Rickettsia* spp. pathogens can be transmitted by ticks, mites, fleas, or lice. They predominantly infect vascular endothelial cells in mammalian hosts, except for *R. akari*, which prefers monocytes and macrophages. However, increasing investigations suggest more robust involvement of monocyte/macrophages than previously believed. Once within the host dermis, CD68^+^ cells (macrophages and dendritic cells) are suspected to be among the first that become infected and disseminate to inevitably infect vascular endothelial cells (Walker et al., 1999; Fournier et al., 2005; Sahni et al., 2019). This leads to vascular instability and microvascular permeability as a result of or in conjunction with rickettsial vasculitis (Mansueto et al., 2012; Påhlson et al., 2021).

Based on the model of *Rickettsia typhi* dissemination in guinea pigs, the likely early and innate role of dendritic cells in both infection and dissemination were initially revealed (Murphy et al., 1978). After intradermal inoculation, viable rickettsiae are still present at the inoculation site up to 5 days. By day 5, the rickettsiae readily disseminate to the draining lymph nodes, but not elsewhere; by day 7, rickettsiae are found in blood and in end organs (spleen and kidney). Interactions between *Rickettsia* spp. and macrophages or dendritic cells are increasingly understood and appear to be critical for infection and induction of innate response that condition subsequent protective adaptive immunity. For example, dendritic cells (DCs) from C3H mice, a susceptible strain, but not from resistant C57BL/6 (B6) mice, are differentially stimulated by *R. conorii* infection. While DCs from both strains differentiate with infection to express increased major histocompatibility complex (MHC) and costimulatory molecules, DCs from B6 animals sustain higher infection densities, higher expression levels of MHC class II, IL12, and increased CD4^+^ T cell priming for gamma interferon (IFNγ) production than do DCs from C3H mice, that develop a suppressive response with a high frequency of Foxp3^+^ T regulatory cells (Fang et al., 2007). Similarly, adoptive transfer of rickettsia-stimulated DCs protect mice from lethal infection through rickettsia-induced differentiation and expression of CD40, CD80, CD86, MHC class II molecules, production of IL2, IL12, and IL23 that together promote T cell IFNγ expression (Jordan et al., 2007). Of interest, *R. conorii* survives infection in the macrophage-like cell line THP-1 whereas the non-pathogenic *R. montanensis* does not, presumably related to the ability of the former to escape the phagolysosome (Curto et al., 2016). Moreover, a key distinction appears to relate to the ability of *R. conorii* to alter macrophage transcriptional programs that include modulated anti-inflammatory gene expression, upregulated pro-survival genes, and expression of distinct transcription factors that result in an M2-like differentiation phenotype. This includes unique events likely exerted through toll-like receptor 3 (TLR3) and toll-like receptor 4(TLR4) signaling that activate nuclear factor-kappaB (NF-κB) and subsequently tumor necrosis factor alpha (TNFα) signaling (Curto et al., 2019a; Curto et al., 2019b). The mechanisms by which this occurs are unclear.

### Innate immunity triggering events

As Gram-negative bacteria, rickettsiae possess a range of potential triggering ligands, including lipopolysaccharide, peptidoglycan and prokaryotic DNA. All of these ligands can be recognized by the innate immune system as pathogen-associated molecular patterns (PAMPs) through host pattern recognition receptors (PRRs) such as membrane-bound Toll-like receptors (TLRs) and the cytoplasmic NOD-like receptors (NLRs) (Schroeder et al., 2016). For the genus *Rickettsia*, recognition can be initiated through TLR4, and molecular adaptors for this pathway involve MyD88 and Toll-receptor-associated activator of interferon (TRIF) that mediate production of inflammatory cytokines as well as components of host cellular immunity (Sahni et al., 2019). Plasma concentrations of proinflammatory cytokines as IL12, IL6, and TNFα are higher in TLR4-competent mice compared with C3H/HeJ mice naturally defective in TLR4 signaling. As a consequence, C3H/HeJ mice develop higher bacterial loads and succumb to otherwise sub-lethal doses of *R. conorii* (Jordan et al., 2008). How much of innate immune recognition occurs at the level of the vascular endothelium is not known. However, TLR4 signaling in DCs stimulated by *R. conorii* activates NK cytotoxicity in the murine model of fatal spotted fever rickettsiosis (Jordan et al., 2009) mediated through MyD88 leading to subsequent NF-κB and IL1β-induced proinflammatory responses (Bechelli et al., 2016). Similarly, live- or heat-killed-*R. akari* can be recognized through TLR2 or TLR4 using transfection-based complementation of TLR2/4-negative HEK293T cells with human TLR2 or TLR4 co-expressed with CD14 and MD-2 resulting in IκBα degradation, NF-κB reporter activation, and IL8 expression (Quevedo-Diaz et al., 2010). Some evidence suggests differential expression of host inflammatory genes with pathogenic *R. conorii* vs. non-pathogenic *R. montanensis*, including the suppression of CD14 required for TLR4 signaling, a potential virulence and survival attribute that correlates with the ability of macrophages and macrophage-like cells to kill non-pathogenic species through lysosomal degradation (Curto et al., 2019a; Kristof et al., 2021). *Asc* knockout mice are more susceptible to *R. australis* infection demonstrating that activation of the inflammasome, likely through NLRP3 in macrophages, is important for protective immunity (Smalley et al., 2016).

### Autophagy and autophagy subversion

Control of intracellular bacteria is often achieved through the induction of autophagy, a cytoplasmic bulk degradation process in which contents are degraded upon fusion with lysosomes for host cell components, or by xenophagy for foreign components, such as intracellular microbes. *R. australis*-infected *Atg5^flox/flox^* Lyz-Cre mice deficient in autophagy in macrophages and granulocytes only, develop lower bacterial loads in liver, spleen, and lung, and higher levels of plasma pro-inflammatory cytokines, including IL1α, IL18, TNFα, and IL6, when compared with *Atg5^flox/flox^* mice in a rickettsia-modified autophagic response (Bechelli et al., 2019; Bechelli et al., 2021). *R. parkeri*, a BSL2 model spotted fever group *Rickettsia* species, employs outer membrane protein B (OmpB) to block ubiquitination of bacterial surface proteins, including OmpA, and subsequent recognition by autophagy receptors to block this process in macrophages (Engström et al., 2019). This hypothesis is confirmed by study of two proteins that *Rickettsia* spp. uses to methylate OmpB lysine residues, protein-lysine methyltransferase enzymes PKMT1 and PKMT2. *R. parkeri pkmt1* and *pkmt2* mutants, compared with *R. parkeri* WT strain in an AG129 (*Ifna/b/gr* ^-/-^) mouse model, succumbed to infection with WT bacteria but not in *pkmt1*- or *pkmt2*-mutants (Engström et al., 2021). Alternative hypotheses that could explain these observations include the possibility that OmpB has deubiquitinase activity, or that OmpB camouflages the bacterial surface itself or recruits other host proteins, but neither is yet demonstrated (Engström et al., 2019).

### Rickettsial adhesion and invasion events

A key to *Rickettsia* spp. evasion of innate immunity is the ability to escape and survive within the host cell cytosol. Bioinformatics-based analyses of rickettsial genomes identify a family of autotransporter genes referred to as surface cell antigen genes (*sca*), including *sca0* through *sca16;* not all Sca proteins/genes are functional. Only Sca0 through Sca5 (Sca0 or OmpA, Sca1, Sca2, Sca3, Sca4, and Sca5 or OmpB) are active, playing roles in adhesion and entry of the host cell (Schroeder et al., 2016; Sahni et al., 2019). But rickettsial adherence is not limited to Sca proteins; two additional proteins are described with rickettsial adhesion, including Adr1 and Adr2 identified in *R. conorii* and *R. prowazekii*, respectively (Schroeder et al., 2016). The earliest identified host receptor involved in rickettsial internalization is Ku70 (subunit of DNA-dependent protein kinase) that interacts with OmpB (Martinez et al., 2005). Other host receptors include α2β1 integrin, fibroblast growth factor receptor-1 (FGFR1), and an unknown protein receptor, perhaps ANXA2 that engages cytosolic Rap guanine nucleotide exchange factor 3 (Epac1), an exchange protein directly activated by cyclic AMP, could have key roles during bacterial adhesion and invasion (Gong et al., 2013; Hillman et al., 2013; Sahni et al., 2017). It is likely that other proteins are involved as well in this process since clathrin and caveolin-2 proteins contribute to endocytosis and rickettsial internalization (Chan et al., 2009). Once the bacteria activate endocytosis, they escape from the early endosome prior to lysosome fusion to enter the cytosol *via* a process mediated by rickettsial phospholipase A2, phospholipase D, hemolysin C, and others (Sahni et al., 2019).

### Animal models of rickettsial innate immunity

Interactions between rickettsiae and hosts, including innate immune responses, are well studied using murine models (Osterloh, 2017; Sahni et al., 2019). C3H/HeN mice are susceptible to a broad range of rickettsiae (Walker et al., 1994; Walker et al., 2000; Londoño et al., 2019). *R. conorii*- and *R. parkeri*-infected C3H/HeN murine models closely mimic human disease and pathology since endothelial cells are the major targets of infection (Walker et al., 1994; Londoño et al., 2019), but require intravenous inoculation to bypass host events in the skin. Alternately, C57BL/6 mice are susceptible to infection only with *R. australis* but highly resistant to *R. conorii* and other rickettsiae (Feng et al., 1993). Regardless, C57BL/6 knockout mice have been important tools for study of the critical host immune system components during rickettsiae infection (Feng et al., 1993; Osterloh, 2017). In this sense, C57BL/6 mice with genotypes that compromise the innate immunity, such as major histocompatibility complex 1 (*Mhc1^-/-^*), perforin (*Prf1^-/-^*), interferon gamma (*Ifng^-/-^*), or Nod-like receptor family pyrin domain containing 3 (*Nlrp3^-/-^*) are more susceptible to *R. australis* infection compared to WT animals (Osterloh, 2017). Rickettsia-infected NK cell-deficient mice are impaired at bacterial clearance, develop early severe liver thrombosis, and have decreased serum IFNγ levels compared with WT mice (Fang et al., 2012). C3H/HeJ mice, which are defective in TLR4 signaling and thereby defective in LPS responses, are more susceptible to *R. conorii* infection compared with the C3H/HeN control strain (Jordan et al., 2008). C57BL/6 *Ifna/b/gr* ^-/-^, and similarly AG129 mice, which share an IFN receptor deficiency genotype, are *R. parkeri* dose-dependent lethal models (Burke et al., 2021). In contrast, individual *Ifnar^−/−^* and *Ifngr^−/−^* mice do not show signs of severe disease even at the highest infectious dose (Burke et al., 2020).

During rickettsial infection, endothelial cells increase i) interactions with leukocytes in an IL1α pathway-dependent manner; ii) secretion of pro-inflammatory cytokines (IL6 and IL8) and expression of chemokines (CCL2, CCL3, CCL4, CCL5, CCL12, CCL19, CCL21, CX3CL1, CXCL1, CXCL9, and CXCL10); iii) expression of adhesion molecules (E-selectin, L-selectin, P-selectin, ICAM1, and VCAM1); iv) altered levels of antioxidant enzymes, accumulation of intracellular reactive oxygen species (ROS), and reduced levels of protective thiols; and, v) activation of host defense expression of TNFα and IFNγ to induce superoxide anion (O_2_^−^) and hydroxyl radicals (OH^−^)(Schroeder et al., 2017; Sahni et al., 2019). All of these changes lead to or end with increased vascular permeability. The proinflammatory signaling NF-κB and mitogen-activated protein kinases (MAPK) pathways are activated during rickettsial infection. Rickettsiae stimulate NF-κB activation *via* the canonical pathway, where IκB (inhibitor of NF-κB) is phosphorylated by IκB kinase (IKK), releasing NF-κB from the NFκB–IκB complex to translocate to the nucleus where it binds to promoters at κB binding sites to increase expression of inflammatory cytokines. The major modules of the MAPK pathways are signal-regulated kinases (ERKs), p38 MAPK, and c-JUN NH2-terminal kinase (JNK), and these can be stimulated independently or constitutively by rickettsial infection (Schroeder et al., 2016).

## Anaplasma phagocytophilum

Human granulocytic anaplasmosis (HGA), caused by infection with *A. phagocytophilum*, an intravacuolar rickettsia that infects predominantly neutrophils in mammals and humans, was first identified in 1990 in a Wisconsin patient who died with a severe febrile illness 2 weeks after a tick bite (Bakken et al., 1994; Chen et al., 1994). HGA is the second most common tick-borne infection in the U.S. and is increasingly recognized as an important and frequent cause of fever after tick bite in many parts of Europe and Asia in areas where *Ixodes* ticks bite humans (Fang et al., 2015; Rosenberg et al., 2018). HGA is clinically variable, but most patients have a moderately severe febrile illness with headache, myalgia, and malaise. Frequent laboratory abnormalities include thrombocytopenia, leukopenia, anemia, and elevated hepatic transaminase levels. Seroepidemiologic data suggests that many infections go unrecognized, especially in endemic areas (Bakken and Dumler, 2015). Severity sufficient for hospitalization is observed in half of symptomatic patients and severe complications include a septic or toxic shock–like syndrome, coagulopathy, atypical pneumonitis/acute respiratory distress syndrome (ARDS), macrophage activation (MAS)/hemophagocytic lymphohistiocytosis (HLH) among others (Bakken and Dumler, 2015).

### Subversion of innate neutrophil responses by *A. phagocytophilum*

*A. phagocytophilum* survives and propagates in neutrophils, abundant, short-lived innate immune phagocytes whose main function is microbial killing. *A. phagocytophilum* released from infected cells are enriched for a population that are infectious (dense core) but metabolically inert. Initial interactions of the bacterium occur by binding of bacterial invasion protein A (AipA), surface protein (Asp14), and outer membrane protein A (OmpA) to mediate optimal entry; *in vivo*, infection is suppressed by antibodies to AipA and Asp14 (Naimi et al., 2020). Adhesion occurs through binding to neutrophil PSGL-1 decorated with α2,3-sialic acid and α1,3-fucose of the sLe^x^ tetrasaccharide (Goodman et al., 1999). Entry depends on the early introduction of the type 4 secretion system effector protein ankyrin A (AnkA) into the neutrophil bound with *A. phagocytophilum*. AnkA then binds to Abl-interactor 1 (Abi-1), an adaptor that mediates an interaction with Abl-1 tyrosine kinase resulting in AnkA phosphorylation. Activation of the WAVE regulatory complex then results in endosome formation, *A. phagocytophilum*’s first perturbation of innate immune control (Lin et al., 2007). Thereafter, the bacterium-containing vacuole accumulates markers of autophagosomes except for the presence of monoubiquitinated proteins and enrichment in cholesterol and Rab proteins. These traffic the vacuole to the trans Golgi network and the endoplasmic reticulum, and as clathrin-independent recycling endosomes, preclude degradation (Truchan et al., 2013; Truchan et al., 2016a; Truchan et al., 2016b). In fact, these attributes are likely a result of *A. phagocytophilum*’s capacity to initiate formation of a multivesicular body as a protected niche in which to propagate and eventually release infectious progeny to sustain infection (Read et al., 2022).

With established infection, *A. phagocytophilum* abrogates key neutrophil functions, including antimicrobial activity, oxidative burst, apoptosis, vascular margination, emigration, and phagocytosis while activating proinflammatory responses including degranulation and cytokine/chemokine production (Dumler, 2012). Infection of neutrophils and HL-60 cells produce striking quantities of chemokines, including IL-8, RANTES, MIP1α, MIP1β, and MCP1, but not other cytokines observed with *in vivo* infection, including IFNγ, IL-10, TNFα, IL1β, or IL4 (Dumler et al., 2000; Martin et al., 2000). Antibody blockade of chemokine receptor 2 (CXCR2) and infection of CXCR2 knockout mice substantially reduces propagation owing to loss of recruitment of ancillary uninfected neutrophils (Scorpio et al., 2004). Moreover, engagement of opsonophagocytic receptors and degranulation are usually accompanied by rapid cell death, but with *A. phagocytophilum*, opsonophagocytic receptor expression is downregulated, precluding a direct antimicrobial effect, and degranulation is protracted, potentially exacerbating inflammation, especially with delayed apoptosis of infected neutrophils (Garyu and Dumler, 2005). These events likely provide a fitness advantage to the bacterium by recruitment and survival of new neutrophil host cells, and increasing the blood concentrations of infected cells that can be acquired by tick bite. The disadvantage of chemokine and other induced proinflammatory responses is collateral inflammatory tissue damage (Scorpio et al., 2005; Browning et al., 2006; Scorpio et al., 2006; Choi et al., 2007).

There are several notable alterations of neutrophil function and physiology observed with *A. phagocytophilum* infection. *A. phagocytophilum* survives its initial encounter by suppression of respiratory burst through epigenetic silencing of *CYBB* (*gp^91phox^*) and *RAC2* (Banerjee et al., 2000; Carlyon and Fikrig, 2006; Garcia-Garcia et al., 2009a; Garcia-Garcia et al., 2009b). When released from the bone marrow, neutrophils are preprogrammed for apoptosis within 12-24h. Delayed apoptosis with *A. phagocytophilum*–infected neutrophils provides a mechanism for expanding microbial populations, and in part relates to p38 MAPK transcriptional activation of *BCL2* family genes and stabilization of the mitochondrial pathway that ultimately prevents procaspase 3 processing (Scaife et al., 2003; Choi et al., 2005; Ge et al., 2005; Lee and Goodman, 2006).

Infection by *A. phagocytophilum* results in significant disruption of other normal neutrophil functions, including diminution of endothelial cell adhesion and transmigration, increased motility of infected cells, degranulation, inhibited respiratory burst, and defective phagocytosis and antimicrobial killing (Choi and Dumler, 2003; Choi et al., 2003; Choi et al., 2004a; Garyu et al., 2005; Bussmeyer et al., 2010; Schaff et al., 2010). *A. phagocytophilum*–infected neutrophils and HL-60 cells are inhibited from binding to HUVEC and brain microvascular endothelial cells, even under conditions of low shear stress (Park et al., 2003). The adhesion defect results from *A. phagocytophilum*-induced degranulation of proinflammatory proteins including metalloproteases that act as sheddases. This results in the loss of neutrophil PSGL-1 and L-selectin, which mediate the critical first step in neutrophil tethering to activated endothelial cell surfaces, despite the rapid mobilization of neutrophil surface β2-integrins (CD11b/CD18) and endothelial cell ICAM1 (CD54) that mediate the second phase of leukocyte arrest on endothelial cells (Choi et al., 2003; Choi et al., 2004a).

Changes in these *A. phagocytophilum*-infected neutrophil functions are in part attributed to altered host cell transcription. Transcriptional profiling of *A. phagocytophilum*-infected neutrophils or terminally differentiated granulocytes demonstrate marked differential expression of a range of neutrophils genes, both up- and down-regulated (Borjesson et al., 2005b; Lee et al., 2008; Dumler et al., 2018). Key pathways impacted by differential gene expression include cytokine-cytokine interactions, apoptosis, cell adhesion molecules, “systemic lupus erythematosus” (T and B cell receptor interactions, complement and coagulation cascades), and toll-like receptor signaling, among others. In addition to its role in *A. phagocytophilum* cell entry, AnkA enters the neutrophil nucleus and binds to multiple genomic loci across every human chromosome, largely enriched in intergenic regions and gene promoters (Dumler et al., 2016). The recognized silencing of *CYBB* upon *A. phagocytophilum* infection results from AnkA binding at the *CYBB* promoter which in turn recruits HDAC1 (Garcia-Garcia et al., 2009b; Rennoll-Bankert et al., 2015). The deacetylated histone H3 tightens DNA coiling, closing chromatin conformation to the exclusion of transcription factors accessible in the native conformation. This was also found present at the promoters of multiple host defense genes that were in turn silenced (Garcia-Garcia et al., 2009a). In fact, HDAC1 activity and expression are increased with infection and critical for *A. phagocytophilum* survival (Rennoll-Bankert et al., 2015). Interestingly, AnkA does not bind DNA in a sequence-dependent manner, but binds to extended enriched stretches of A, T, and C nucleotides on a single strand that identify matrix attachment regions (MARs) (Garcia-Garcia et al., 2009b). AT richness is a common feature of unstable base unpairing regions (BURs) that dissociate when placed under negative superhelical stress. BURs are found within MARs, specialized DNA structures that serve as attachment sites for nuclear matrix proteins such as lamins, scaffold attachment factor-1 and the special AT binding protein-1 (SATB1) known to organize nuclear chromatin for tissue-specific gene expression, chromatin accessibility and long-range chromatin conformational modifications (Galande and Kohwi-Shigematsu, 2000; Hawkins et al., 2001; Yasui et al., 2002; Cai et al., 2003; Cai et al., 2006). AnkA binds known and predicted MARs and intergenic regions established to interact with lamina associated domains that partition DNA into an inactive configuration within the nuclear lamina (Dumler et al., 2016). Whether these regions could also be released from the nuclear lamina for coordinated expression within transcriptional factories coalesced conceivably around AnkA in order to dramatically alter overall cellular transcriptional programs is an area of active investigation.

### Innate immune recognition of *A. phagocytophilum*

Innate immune recognition of *A. phagocytophilum* is complex and many cellular responses with infection were discovered using animal models, including mice, horses, and dogs. Immune competent mice do not exhibit clinical signs of infection but develop histopathology characteristic of human and equine infections (Bunnell et al., 1999; Lepidi et al., 2000; Martin et al., 2000). In contrast to murine models, infection of horses nearly precisely mimics human infection, including the spectrum of clinical severity (Pusterla et al., 2000; Davies et al., 2011). While the initial interactions of *A. phagocytophilum* with neutrophils are well-studied, interactions with other immune cells that initiate innate immune responses, such as DCs, are less well defined. *A. phagocytophilum* differs from many other Gram negative bacteria in that it lacks both LPS and peptidoglycan, ligands for many common pattern recognition receptors. Unlike successful propagation in granulocytes and differentiated neutrophils, *A. phagocytophilum* does not survive in differentiated macrophages (Heimer et al., 1997). Alternate mechanisms for initiating innate immune cellular responses, such as NF-κB nuclear translocation, include *A. phagocytophilum* interactions with TLR2 but not TLR4 (Choi et al., 2004b). An analysis of NF-κB activation implicates both canonical and non-canonical signaling and a potential variety of microbial and cellular signals for recognition (Dumler et al., 2020a). A role for *A. phagocytophilum* glycolipids or lipids in recognition and inflammatory activation of NK and NKT cells is supported by histopathologic studies in murine models (Scorpio et al., 2005; Scorpio et al., 2006; Choi et al., 2007); in fact, polar lipid membrane extracts from *A. phagocytophilum* activate proliferation in splenocytes from naïve animals, (Choi and Dumler, 2007). Similarly, the pattern recognition receptor NLRC4 and inflammasome activation of mononuclear phagocytes occurs *via* eicosanoid and prostaglandin E2 *via* the EP3 receptor and is in part dependent on receptor-interacting serine/threonine-protein kinase 2 (RIPK2), implicating multiple mechanisms for immune recognition and response (Chen et al., 2012; Wang et al., 2016a; Wang et al., 2016b).

Once activated, a range of immune signaling mechanisms are directly involved in the generation of proinflammatory responses that lead to tissue injury, but that do not affect pathogen replication or load (Von Loewenich et al., 2004; Scorpio et al., 2006). This includes signaling or effector pathways through IFNγ, IL10, NOX2 (*Cybb*), NOS2, TNF, MyD88, TLR2, and others. Similarly, NK and NKT, but not CD8 or CD4 cells have a role in enhanced inflammatory tissue injury in the absence of changes in bacterial load (Choi et al., 2007; Birkner et al., 2008).

The mechanisms of pancytopenia with HGA are unclear, but some evidence points to pathogen activation of macrophages by proinflammatory cytokines, such as IFNγ in tissue sequestration or destruction (Borjesson et al., 2001; Borjesson et al., 2005a; Dumler et al., 2007; Davies et al., 2011; Dumler, 2012; Scorpio et al., 2018). *A. phagocytophilum* paradoxically activates innate immune responses that contribute to tissue injury and disease despite its ability to avoid killing by these mechanisms (Von Loewenich et al., 2004; Browning et al., 2006; Scorpio et al., 2006; Choi et al., 2007; Birkner et al., 2008; Choi et al., 2014). Immune responses in mouse models demonstrate the critical role of IFNγ in induction of severe inflammatory histopathology, even in the absence of significant bacterial loads (Martin et al., 2000; Martin et al., 2001). Typically, intracellular bacteria are controlled after eliciting cell-mediated immune responses characterized by Th1 cytokines, IL12, and IFNγ, and in fact, humans with HGA develop very high levels of the Th1 cytokines IFNγ and IL12 during active infection (Dumler et al., 2000; Dumler et al., 2007). Mice also exhibit a substantial increase in the plasma IFNγ level with infection, and the lack of *A. phagocytophilum* growth restriction in immunocompromised mouse strains is consistent with an important role for acquired immunity in controlling bacterial burdens and complete bacterial killing (Martin et al., 2000; Von Loewenich et al., 2004; Birkner et al., 2008). In addition to control of *A. phagocytophilum* infection, IFNγ production in the murine HGA model is a major contributor to inflammatory tissue injury since histopathologic lesions in *Ifng* knockout mice are completely abrogated despite a marked increase in the pathogen load, implying that most histopathologic injury that belies disease is immune-mediated (Martin et al., 2000).

### STAT1 and IFNγ signaling

Signal transducer and activator of transcription 1 (STAT1) mediates many of the biological functions of both type I (IFNα/β) and type II (IFNγ) interferons and is important in host innate and adaptive immune responses to viruses and other intracellular pathogens, especially intracellular bacteria. While IFNα/β depends on the presence of the canonical signaling molecules STAT1, STAT2, and interferon regulatory factor 9 (IRF9), IFNγ signaling is mostly dependent on STAT1. While IFNα/β-dependent mechanisms interfere with virus replication, in contrast, IFNγ mediates resistance against intracellular bacteria and protozoa, primarily through its ability to activate macrophages. The loss of STAT1 function in humans (or mice) results in a dramatically increased susceptibility to viral, bacterial, and protozoan infections (Hofer et al., 2012; Kernbauer et al., 2012; Rauch et al., 2013). *A. phagocytophilum* infection-induced IFNγ signaling leads to phosphorylation of Stat1 in mice (Choi and Dumler, 2013), and dexamethasone suppression of STAT1 activity in *A. phagocytophilum*-infected horses reduces inflammatory signaling and disease severity (Davies et al., 2011).

With the murine model of *A. phagocytophilum* infection, IFNγ and its signaling through phosphorylation, homodimerization and nuclear translocation of STAT1, leads to macrophage activation and activated macrophages represent a dominant effector phase of the innate immune response and subsequently leads to host tissue injury and disease (Akkoyunlu and Fikrig, 2000; Martin et al., 2001). In contrast, deficiency of IL10 (IL10 knockout mice) that signals *via* STAT3, leads to restriction of microbial growth but marked enhancement of inflammatory tissue injury with extensive necrosis and pyroptosis, presumably owing to its role in counterbalancing IFNγ effects (Martin et al., 2001).

The absence of STAT1, the major proinflammatory signaling pathway for IFNγ, converts the subclinical infection phenotype universally observed with *A. phagocytophilum* mouse models to one with severe clinical signs (reduced activity, piloerection, crouching, dehydration, and weight loss), dense infiltration of inflammatory cells, and robust proinflammatory cytokine/chemokine responses compared with WT controls (Choi et al., 2014a). Although STAT1 is not essential to eventual resolution of the infection (Birkner et al., 2008), it plays an important role in innate immune protection and provides evidence of an unexplained and potentially novel STAT1-dependent homeostatic mechanism that dampens host inflammatory and immune organ/tissue damage with *A. phagocytophilum* infection (Choi et al., 2014). Lacking STAT1, infection triggers a marked immune response discoordination with hypercytokinemia, significantly more inflammation, and increased bacterial loads, as has been observed with bacterial, protozoan, and viral infections (Lieberman et al., 2004; Rayamajhi et al., 2010; Kernbauer et al., 2012). This observation also underscores the necessity of macrophage activation mediated by IFNγ signaling *via* STAT1 for both control of pathogen loads and coordination of appropriate inflammatory response.

Inflammatory and innate immune signaling with *A. phagocytophilum* infection is thought to be initiated in one of several ways. Stimulation *via* TLR2 and perhaps other PRRs drives NF-κB and proinflammatory gene expression (Choi et al., 2004b; Dumler et al., 2020b). In fact, most of the chemokines and cytokines upregulated in *Stat1* knockout mice after infection are driven by NF-κB that binds to κB sites in promoter sequences at gene loci that could easily be explained by TLR activation (Choi et al., 2014; Dumler et al., 2020a). Similarly, loss of the MyD88 adapter for TLR2, such as with infection in *Myd88* knockout mice, also diminishes inflammatory phenotype without affecting bacterial loads (Scorpio et al., 2006).

The biological consequences of proinflammatory signaling *via* NF-κB and IFNγ/STAT1 have many redundancies, but the latter allows predominantly for a process by which macrophages become activated for effector responses such as increased microbial killing (Myers and Gottschalk, 2022). Similarly, type I interferons can also promote inflammatory response and antiviral effects. However, the loss of STAT1 signaling leads to reduced signal capacity because both type I (IFNα and IFNβ) and type II (IFNγ) interferons use STAT1, either as hetero- or homodimers, for canonical activation of downstream transcription (Meyts and Casanova, 2021). An important target of both type I and type II interferons is upregulation of inducible nitric oxide synthase (iNOS) that generates nitric oxide, an important antimicrobial effector with macrophage activation. It has been shown that *Stat1* knockout mice are unable to produce detectable iNOS, likely resulting in the loss of 2 of the 3 major transcriptional activators (Samardzic et al., 2001). In turn, its absence confirms the loss of macrophage activation despite a more severe inflammatory process that, even with greater tissue severity compared with WT animals, lacks the degrees of necrosis and pyroptosis observed with infection in the absence of IL10 and extreme macrophage activation (Martin et al., 2001).

Overall, *Stat1* knockout mice develop more severe disease and Th1-skewed acute inflammation in response to *A. phagocytophilum* infection compared with WT mice. STAT1 is fundamentally important for host defenses against *A. phagocytophilum* infection, leading to macrophage activation and inevitably, markedly improved control of infection (Choi et al., 2014). Absence of STAT1 results in hypercytokinemia and a discoordinated, non-protective immune response triggered by *A. phagocytophilum* infection, further suggesting that severity of *A. phagocytophilum* infection could in part be linked to polymorphisms of STAT1 in humans (Boisson-Dupuis et al., 2012).

While it is likely that a combination of innate immune and inflammation-related factors contributes to disease phenotype, they also likely work to control the pathogen. However, specific antibody alone can exert control over *A. phagocytophilum* and other Anaplasmataceae infections (Sun et al., 1997), and STAT1 deficiency does not diminish antibody response.

### NK and NKT lymphocyte cytotoxicity

NK cells and NKT cytotoxic cells are activated and participate in the development of histopathologic lesions with *A. phagocytophilum* infection (Choi et al., 2007; Scorpio et al., 2018). The niche occupied by obligate intracellular bacteria also presents a significant challenge to host immunity. NK1.1-expressing cells, either NK or NKT, are innate immune cells that can produce abundant IFNγ. In an *A. phagocytophilum*-infected mouse model, all NK1.1^+^ and activated (FasL-expressing) NK1.1^+^ cells comprise a significantly greater proportion of *ex vivo* splenocytes on days 4 and 7 of infection (Choi et al., 2007). The differential expansion of NK and activated FasL-expressing NK cells suggests that they play a role in the differential histopathologic inflammatory severity (Walker and Dumler, 2015). Diminution of hepatic pathology in NK- and NKT-deficient mice confirms the role (Choi et al., 2007) where peak hepatic inflammation occurs marginally earlier than the expansion of NK and NKT lymphocytes, and both types of cells comprise an exceedingly small proportion of splenocytes on day 2, at the time of peak inflammation and plasma IFNγ levels.

Another potentially significant source of IFNγ during early phases of infection includes NKT cells, which are implicated in infections by *Ehrlichia muris* (Stevenson et al., 2008). A role for NKT cells was supported by significant activation-induced loss of NK1.1^+^ (including both NK and NKT cells) and NK1.1/TCR-expressing (NKT) splenic cells in infected but not uninfected animals when hepatic histopathologic injury accelerates (Choi et al., 2007). Paradoxically, *ex vivo* stimulation of dendritic cells with *A. phagocytophilum* does not result in a significant increase in NKT cell intracellular IFNγ production, while NK cells are equally activated for IFNγ production as with dendritic cell stimulation by the TLR2 ligand, Pam3Cys (Scorpio et al., 2018). The absolute source of IFNγ that drives the inflammatory response is still not determined.

### *A. phagocytophilum*, macrophage activation syndrome (MAS) and hemophagocytic lymphohistiocytosis (HLH)

The immune response mechanisms by which *A. phagocytophilum* clearance occurs depends on CD4 T cells in the absence of perforin, Fas/FasL, major Th1 cytokines such as IL12, IFNγ, CCL2 (MCP1), and a range of innate immune signaling molecules and effectors. This is novel finding for an intracellular pathogen, for which CD4 T cell-, IL12-, and IFNγ-dependent immunity is typically required to clear bacteria (Von Loewenich et al., 2004; Birkner et al., 2008). How CD4 T cells accomplish *A. phagocytophilum* clearance is still not understood, although some evidence suggests IFNγ activates neutrophils in an iNOS-independent process (Gussmann et al., 2017). During infection, many innate and adaptive immune pathways are activated, but few impact microbial burden (Martin et al., 2000; Martin et al., 2001; Scorpio et al., 2004; Von Loewenich et al., 2004; Browning et al., 2006; Scorpio et al., 2006; Choi et al., 2007; Birkner et al., 2008; Choi et al., 2014). It is clear that neutrophil infection contributes to disease by induction of proinflammatory responses and lack of antimicrobial killing. Key protective mechanisms are initiated *via* macrophages and other APCs that do not sustain infection but contribute to the proinflammatory response, severe hypercytokinemia, and disease severity. Aspects of human disease, including MAS and HLH, are mimicked in mouse and equine models of human granulocytic anaplasmosis (Dumler et al., 2007; Tsiodras et al., 2017; Yi et al., 2017; Camacci et al., 2018b).

MAS and HLH are related disorders that have either a genetic basis or an infectious trigger (Crayne et al., 2019; Knaak et al., 2020; Andersson, 2021). Both are cytokine-driven and characterized by progressive fever, shock, organ failure, pancytopenia, liver dysfunction, and coagulopathy, and usually attributed to excessive IFNγ production. Genetic forms of HPS result from mutations of genes encoding proteins involved in signaling perforin or granzyme delivery for cytolysis of target cells resulting in impaired cytotoxic lymphocyte (CTL) function, often among NK cells (Schulert et al., 2016; Schulert and Cron, 2020). Similarly, infection-associated HLH is characterized by defects in CTLs, either by NK lymphopenia or NK cell defects in perforin delivery, although HLH has been observed in humans and animal models with defects in CD8 T cells as well (Steen et al., 2023). The explanation for the relentless progression with HLH and MAS is that the APC-cytotoxic cell synapse through TCR–MHC class I interaction leads to activation of CTLs and production of IL12, IL18, IL15, and IL2, resulting in lymphoproliferation and IFNγ generation. IFNγ activates macrophage effector production (nitric oxide, reactive oxygen species, TNFα, phagocytosis). However, the inability of CTLs to deliver perforin to the APC presenting a cognate ligand frees the cascade from regulation by loss of cytolysis of the APC, exacerbating disease due to unremitting APC activation and cytokine stimulation (Steen et al., 2023). This supports the concept of impaired cytotoxicity of innate and adaptive immune CTLs that are unable to exert homeostatic control of APCs following antigen processing and MHC class I expression to these CTLs. While IFNγ is readily detected during *in vivo A. phagocytophilum* infections in humans as well as in murine or equine models, the relative lack of its expression in CTLs after exposure to APCs that should otherwise present microbial targets for activation suggests that it originates from other sources and that the functional defect could instead reside within the APC (Scorpio et al., 2018). While not the classical MAS paradigm, *A. phagocytophilum* infection and the ongoing production of IFNγ by other cells coupled with the lack of effective feedback cytotoxicity by classical CTLs would be equivalent.

### Arthropod saliva can assist with pathogen transmission

Since spotted fever group *Rickettsia* and *A. phagocytophilum* are mainly transmitted by tick bites, there is a critical interest to understand the role of tick saliva in pathogen transmission. Intradermal infection of C3H/HeN mice with *R. parkeri*, when inoculated with *Amblyomma maculatum* tick saliva, have less dermal infiltration by neutrophils and macrophages at the inoculation site after 6 and 24h, respectively, than those without tick saliva inoculation, yet these changes do not alter bacterial concentrations (Banajee et al., 2016). In contrast, the tick protein sialostatin L2 which binds to the cellular receptor annexin A2 and inhibits inflammasome activation *via* NLRC4, renders annexin A2-deficient mice more susceptible to *A. phagocytophilum* infection. Translational support for this concept is shown with recombinant sialostatin L2 binding better to annexin A2 in human serum from patients with acute *A. phagocytophilum* infections compared to uninfected controls (Wang et al., 2016b).

## Conclusions

Investigators of intracellular bacteria and the diseases they cause in humans often address unanswered questions by investigating processes shared with other bacteria or bacterial processes, or by investigating differences that allow the unique niche to become occupied. This review describes two bacteria that share many innate immune responses elicited by infection. However, some important differences exist, including (**Table 1**): 1) *Rickettsia* spp. likely infect CD68^+^ macrophages and dendritic cells initially, although the ultimate targets are endothelial cells; TLR4 is important for recognition with initial infection and animals deficient in this signaling pathway are more susceptible to infection, and infection can alter transcriptional programs in these cells to potentially benefit propagation and influence disease severity; disease results from both direct effects of the pathogen on critical vascular barriers in organs and the ensuing inflammatory responses. 2) *A. phagocytophilum* infects only cells of the myeloid granulocyte lineages, especially neutrophils, but also eosinophils, basophils, and possibly mast cells; *A. phagocytophilum* infection has the capacity to cause severe disease in humans while in some animal reservoirs, persistent subclinical states are maintained; TLR2 is important for initial infection recognition in antigen-presenting cells; the secreted effector, AnkA, moonlights to assist rapid endocytosis, and reprograms neutrophil functions to benefit microbial survival; disease in anaplasmosis is not directly related to pathogen burden, but to the triggering of potentially detrimental and poorly-regulated host inflammatory and immune-mediated processes.

Recent investigations provide important data on the range of functional changes among infected host cells and identify several compelling targets for study of fundamental pathogenetic processes. There are still important areas that need intense study including the bacterial triggers of host innate and inflammatory response and the molecular and cellular mechanisms by which rickettsiae and *A. phagocytophilum* influence cell functions that ultimately result in injury to host cells, tissues, and organs. Due to the lack of vaccines and immunotherapeutics, continued investigation is required to develop strategies that will either minimize or protect against infections by these obligate intracellular bacteria to minimize their many clinical consequences.

## Author contributions

All authors listed have made a substantial, direct, and intellectual contribution to the work and approved it for publication.
